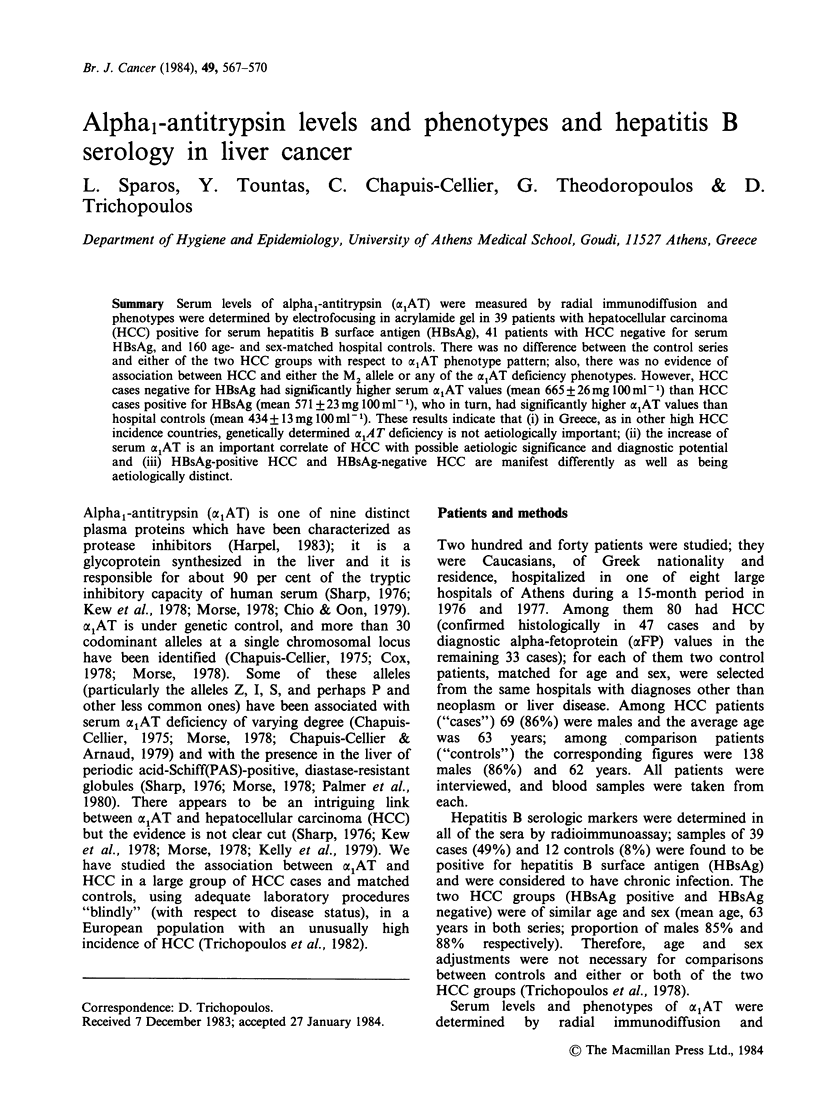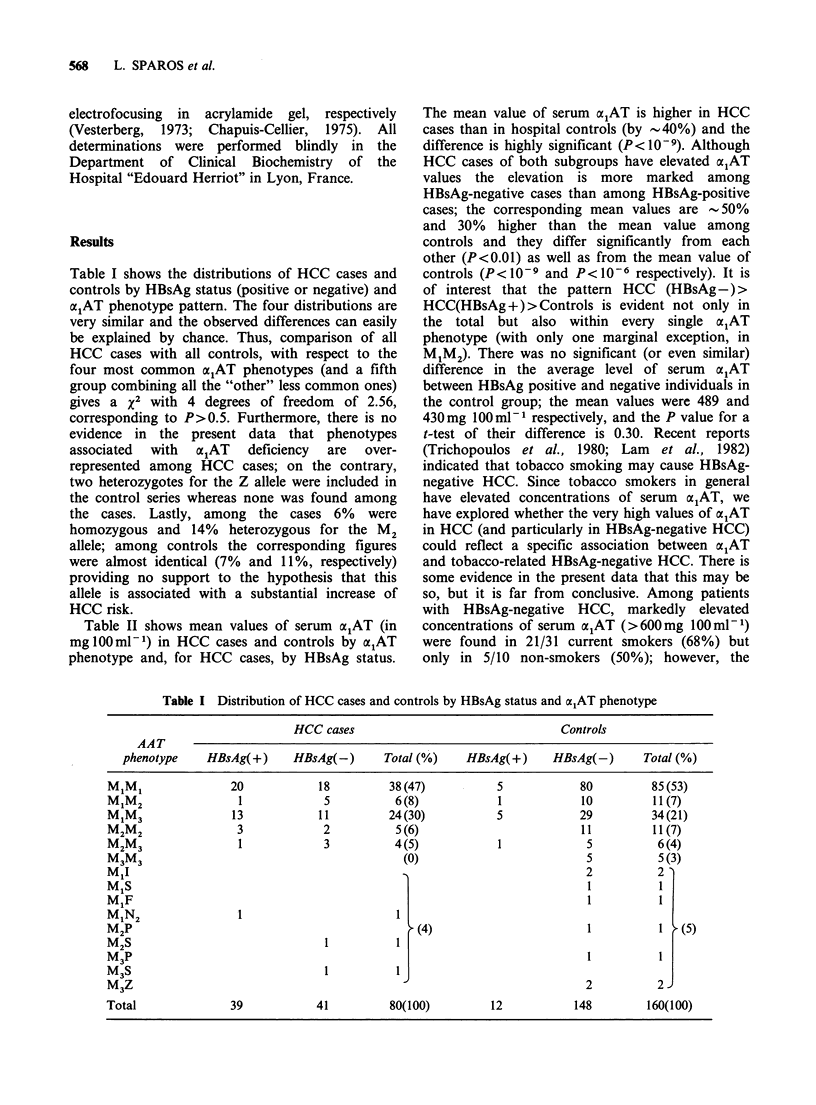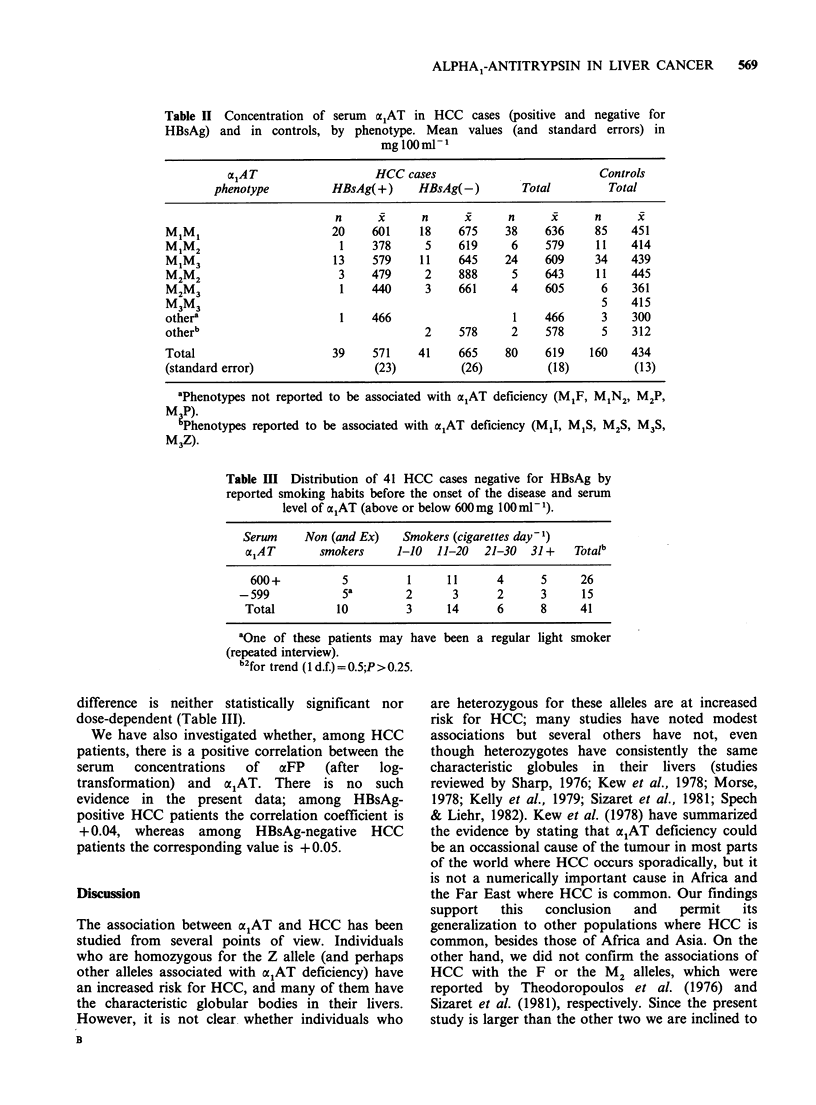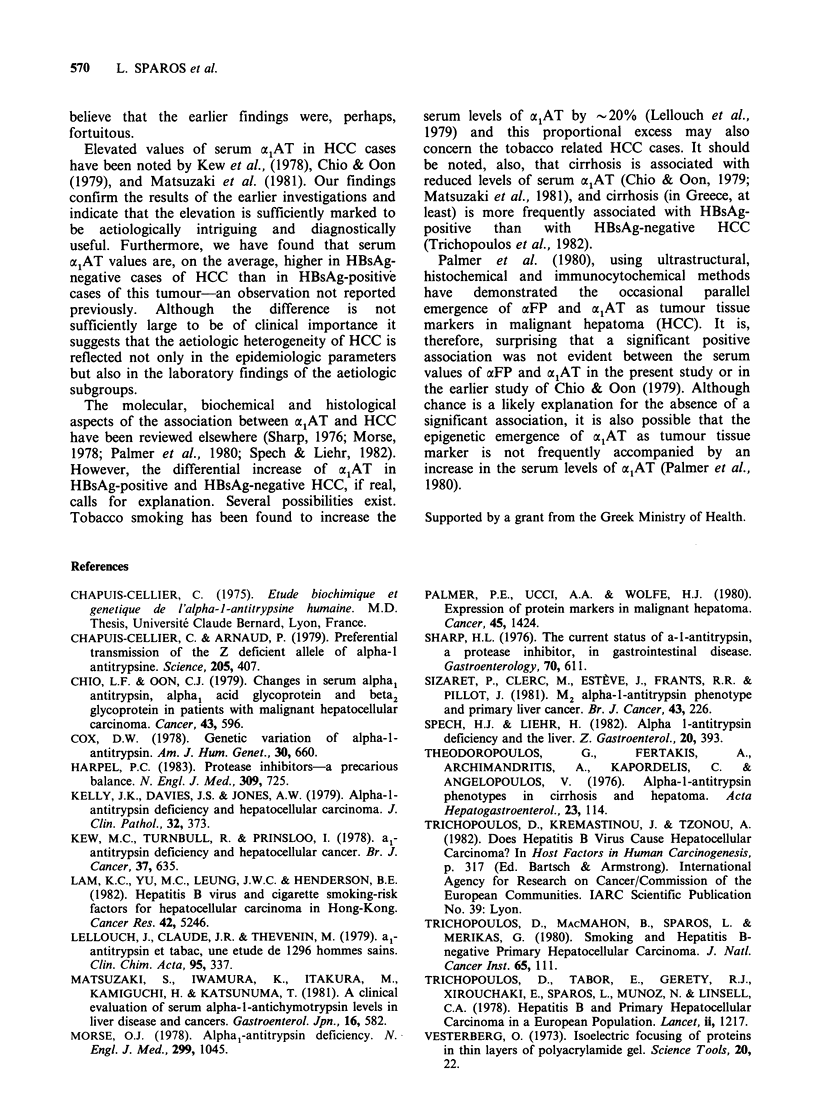# Alpha 1-antitrypsin levels and phenotypes and hepatitis B serology in liver cancer.

**DOI:** 10.1038/bjc.1984.90

**Published:** 1984-05

**Authors:** L. Sparos, Y. Tountas, C. Chapuis-Cellier, G. Theodoropoulos, D. Trichopoulos

## Abstract

Serum levels of alpha 1-antitrypsin (alpha 1 AT) were measured by radial immunodiffusion and phenotypes were determined by electrofocusing in acrylamide gel in 39 patients with hepatocellular carcinoma (HCC) positive for serum hepatitis B surface antigen (HBsAg), 41 patients with HCC negative for serum HBsAg, and 160 age- and sex-matched hospital controls. There was no difference between the control series and either of the two HCC groups with respect to alpha 1 AT phenotype pattern; also, there was no evidence of association between HCC and either the M2 allele or any of the alpha 1 AT deficiency phenotypes. However, HCC cases negative for HBsAg had significantly higher serum alpha 1 AT values (mean 665 +/- 26 mg 100 ml-1) than HCC cases positive for HBsAg (mean 571 +/- 23 mg 100 ml-1), who in turn, had significantly higher alpha 1 AT values than hospital controls (mean 434 +/- 13 mg 100 ml-1). These results indicate that in Greece, as in other high HCC incidence countries, genetically determined alpha 1 AT deficiency is not aetiologically important; the increase of serum alpha 1 AT is an important correlate of HCC with possible aetiologic significance and diagnostic potential and HBsAg-positive HCC and HBsAg-negative HCC are manifest differently as well as being aetiologically distinct.


*Br. J. Cancer* (1984), **49**, 567–570

### Alpha$_1$-antitrypsin levels and phenotypes and hepatitis B serology in liver cancer

L. Sparos, Y. Tountas, C. Chapuis-Cellier, G. Theodoropoulos & D. Trichopoulos

*Department of Hygiene and Epidemiology, University of Athens Medical School, Goudi, 11527 Athens, Greece*

**Summary** Serum levels of alpha$_1$-antitrypsin ($\alpha_1$AT) were measured by radial immunodiffusion and phenotypes were determined by electrofocusing in acrylamide gel in 39 patients with hepatocellular carcinoma (HCC) positive for serum hepatitis B surface antigen (HBsAg), 41 patients with HCC negative for serum HBsAg, and 160 age- and sex-matched hospital controls. There was no difference between the control series and either of the two HCC groups with respect to $\alpha_1$AT phenotype pattern; also, there was no evidence of association between HCC and either the $M_2$ allele or any of the $\alpha_1$AT deficiency phenotypes. However, HCC cases negative for HBsAg had significantly higher serum $\alpha_1$AT values (mean $665 \pm 26$ mg 100 ml$^{-1}$) than HCC cases positive for HBsAg (mean $571 \pm 23$ mg 100 ml$^{-1}$), who in turn, had significantly higher $\alpha_1$AT values than hospital controls (mean $434 \pm 13$ mg 100 ml$^{-1}$). These results indicate that (i) in Greece, as in other high HCC incidence countries, genetically determined $\alpha_1 AT$ deficiency is not aetiologically important; (ii) the increase of serum $\alpha_1$AT is an important correlate of HCC with possible aetiologic significance and diagnostic potential and (iii) HBsAg-positive HCC and HBsAg-negative HCC are manifest differently as well as being aetiologically distinct.

Alpha$_1$-antitrypsin ($\alpha_1$AT) is one of nine distinct plasma proteins which have been characterized as protease inhibitors (Harpel, 1983); it is a glycoprotein synthesized in the liver and it is responsible for about 90 per cent of the tryptic inhibitory capacity of human serum (Sharp, 1976; Kew *et al.*, 1978; Morse, 1978; Chio & Oon, 1979). $\alpha_1$AT is under genetic control, and more than 30 codominant alleles at a single chromosomal locus have been identified (Chapuis-Cellier, 1975; Cox, 1978; Morse, 1978). Some of these alleles (particularly the alleles Z, I, S, and perhaps P and other less common ones) have been associated with serum $\alpha_1$AT deficiency of varying degree (Chapuis-Cellier, 1975; Morse, 1978; Chapuis-Cellier & Arnaud, 1979) and with the presence in the liver of periodic acid-Schiff(PAS)-positive, diastase-resistant globules (Sharp, 1976; Morse, 1978; Palmer *et al.*, 1980). There appears to be an intriguing link between $\alpha_1$AT and hepatocellular carcinoma (HCC) but the evidence is not clear cut (Sharp, 1976; Kew *et al.*, 1978; Morse, 1978; Kelly *et al.*, 1979). We have studied the association between $\alpha_1$AT and HCC in a large group of HCC cases and matched controls, using adequate laboratory procedures "blindly" (with respect to disease status), in a European population with an unusually high incidence of HCC (Trichopoulos *et al.*, 1982).

Correspondence: D. Trichopoulos.
Received 7 December 1983; accepted 27 January 1984.

### Patients and methods

Two hundred and forty patients were studied; they were Caucasians, of Greek nationality and residence, hospitalized in one of eight large hospitals of Athens during a 15-month period in 1976 and 1977. Among them 80 had HCC (confirmed histologically in 47 cases and by diagnostic alpha-fetoprotein ($\alpha$FP) values in the remaining 33 cases); for each of them two control patients, matched for age and sex, were selected from the same hospitals with diagnoses other than neoplasm or liver disease. Among HCC patients ("cases") 69 (86%) were males and the average age was 63 years; among comparison patients ("controls") the corresponding figures were 138 males (86%) and 62 years. All patients were interviewed, and blood samples were taken from each.

Hepatitis B serologic markers were determined in all of the sera by radioimmunoassay; samples of 39 cases (49%) and 12 controls (8%) were found to be positive for hepatitis B surface antigen (HBsAg) and were considered to have chronic infection. The two HCC groups (HBsAg positive and HBsAg negative) were of similar age and sex (mean age, 63 years in both series; proportion of males 85% and 88% respectively). Therefore, age and sex adjustments were not necessary for comparisons between controls and either or both of the two HCC groups (Trichopoulos *et al.*, 1978).

Serum levels and phenotypes of $\alpha_1$AT were determined by radial immunodiffusion and

© The Macmillan Press Ltd., 1984

electrofocusing in acrylamide gel, respectively (Vesterberg, 1973; Chapuis-Cellier, 1975). All determinations were performed blindly in the Department of Clinical Biochemistry of the Hospital "Edouard Herriot" in Lyon, France.

### Results

Table I shows the distributions of HCC cases and controls by HBsAg status (positive or negative) and $\alpha_1$AT phenotype pattern. The four distributions are very similar and the observed differences can easily be explained by chance. Thus, comparison of all HCC cases with all controls, with respect to the four most common $\alpha_1$AT phenotypes (and a fifth group combining all the "other" less common ones) gives a $\chi^2$ with 4 degrees of freedom of 2.56, corresponding to $P>0.5$. Furthermore, there is no evidence in the present data that phenotypes associated with $\alpha_1$AT deficiency are over-represented among HCC cases; on the contrary, two heterozygotes for the Z allele were included in the control series whereas none was found among the cases. Lastly, among the cases 6% were homozygous and 14% heterozygous for the $M_2$ allele; among controls the corresponding figures were almost identical (7% and 11%, respectively) providing no support to the hypothesis that this allele is associated with a substantial increase of HCC risk.

Table II shows mean values of serum $\alpha_1$AT (in mg 100 ml$^{-1}$) in HCC cases and controls by $\alpha_1$AT phenotype and, for HCC cases, by HBsAg status. The mean value of serum $\alpha_1$AT is higher in HCC cases than in hospital controls (by ~40%) and the difference is highly significant ($P<10^{-9}$). Although HCC cases of both subgroups have elevated $\alpha_1$AT values the elevation is more marked among HBsAg-negative cases than among HBsAg-positive cases; the corresponding mean values are ~50% and 30% higher than the mean value among controls and they differ significantly from each other ($P<0.01$) as well as from the mean value of controls ($P<10^{-9}$ and $P<10^{-6}$ respectively). It is of interest that the pattern HCC (HBsAg−)> HCC(HBsAg+)>Controls is evident not only in the total but also within every single $\alpha_1$AT phenotype (with only one marginal exception, in $M_1M_2$). There was no significant (or even similar) difference in the average level of serum $\alpha_1$AT between HBsAg positive and negative individuals in the control group; the mean values were 489 and 430 mg 100 ml$^{-1}$ respectively, and the $P$ value for a $t$-test of their difference is 0.30. Recent reports (Trichopoulos *et al.*, 1980; Lam *et al.*, 1982) indicated that tobacco smoking may cause HBsAg-negative HCC. Since tobacco smokers in general have elevated concentrations of serum $\alpha_1$AT, we have explored whether the very high values of $\alpha_1$AT in HCC (and particularly in HBsAg-negative HCC) could reflect a specific association between $\alpha_1$AT and tobacco-related HBsAg-negative HCC. There is some evidence in the present data that this may be so, but it is far from conclusive. Among patients with HBsAg-negative HCC, markedly elevated concentrations of serum $\alpha_1$AT (>600 mg 100 ml$^{-1}$) were found in 21/31 current smokers (68%) but only in 5/10 non-smokers (50%); however, the

**Table I** Distribution of HCC cases and controls by HBsAg status and $\alpha_1$AT phenotype

| *AAT phenotype* | *HCC cases* | | | *Controls* | | |
|---|---|---|---|---|---|---|
| | *HBsAg*(+) | *HBsAg*(−) | *Total* (%) | *HBsAg*(+) | *HBsAg*(−) | *Total* (%) |
| $M_1M_1$ | 20 | 18 | 38 (47) | 5 | 80 | 85 (53) |
| $M_1M_2$ | 1 | 5 | 6 (8) | 1 | 10 | 11 (7) |
| $M_1M_3$ | 13 | 11 | 24 (30) | 5 | 29 | 34 (21) |
| $M_2M_2$ | 3 | 2 | 5 (6) | | 11 | 11 (7) |
| $M_2M_3$ | 1 | 3 | 4 (5) | 1 | 5 | 6 (4) |
| $M_3M_3$ | | | (0) | | 5 | 5 (3) |
| $M_1I$ | | | | | 2 | 2 |
| $M_1S$ | | | | | 1 | 1 |
| $M_1F$ | | | | | 1 | 1 |
| $M_1N_2$ | 1 | | 1 | | | |
| $M_2P$ | | | (4) | | 1 | 1 (5) |
| $M_2S$ | | 1 | 1 | | | |
| $M_3P$ | | | | | 1 | 1 |
| $M_3S$ | | 1 | 1 | | | |
| $M_3Z$ | | | | | 2 | 2 |
| Total | 39 | 41 | 80 (100) | 12 | 148 | 160 (100) |

**Table II** Concentration of serum $\alpha_1$AT in HCC cases (positive and negative for HBsAg) and in controls, by phenotype. Mean values (and standard errors) in mg 100 ml$^{-1}$

| *$\alpha_1$AT phenotype* | *HCC cases HBsAg(+)* | | *HBsAg(−)* | | *Total* | | *Controls Total* | |
|---|---|---|---|---|---|---|---|---|
| | *n* | $\bar{x}$ | *n* | $\bar{x}$ | *n* | $\bar{x}$ | *n* | $\bar{x}$ |
| $M_1M_1$ | 20 | 601 | 18 | 675 | 38 | 636 | 85 | 451 |
| $M_1M_2$ | 1 | 378 | 5 | 619 | 6 | 579 | 11 | 414 |
| $M_1M_3$ | 13 | 579 | 11 | 645 | 24 | 609 | 34 | 439 |
| $M_2M_2$ | 3 | 479 | 2 | 888 | 5 | 643 | 11 | 445 |
| $M_2M_3$ | 1 | 440 | 3 | 661 | 4 | 605 | 6 | 361 |
| $M_3M_3$ | | | | | | | 5 | 415 |
| other[a] | 1 | 466 | | | 1 | 466 | 3 | 300 |
| other[b] | | | 2 | 578 | 2 | 578 | 5 | 312 |
| Total | 39 | 571 | 41 | 665 | 80 | 619 | 160 | 434 |
| (standard error) | | (23) | | (26) | | (18) | | (13) |

[a]Phenotypes not reported to be associated with $\alpha_1$AT deficiency ($M_1F$, $M_1N_2$, $M_2P$, $M_3P$).
[b]Phenotypes reported to be associated with $\alpha_1$AT deficiency ($M_1I$, $M_1S$, $M_2S$, $M_3S$, $M_3Z$).

**Table III** Distribution of 41 HCC cases negative for HBsAg by reported smoking habits before the onset of the disease and serum level of $\alpha_1$AT (above or below 600 mg 100 ml$^{-1}$).

| *Serum $\alpha_1$AT* | *Non (and Ex) smokers* | *Smokers (cigarettes day$^{-1}$) 1–10* | *11–20* | *21–30* | *31+* | *Total[b]* |
|---|---|---|---|---|---|---|
| 600+ | 5 | 1 | 11 | 4 | 5 | 26 |
| −599 | 5[a] | 2 | 3 | 2 | 3 | 15 |
| Total | 10 | 3 | 14 | 6 | 8 | 41 |

[a]One of these patients may have been a regular light smoker (repeated interview).
[b]$^{2}$for trend (1 d.f.) $=0.5; P>0.25$.

difference is neither statistically significant nor dose-dependent (Table III).

We have also investigated whether, among HCC patients, there is a positive correlation between the serum concentrations of $\alpha$FP (after log-transformation) and $\alpha_1$AT. There is no such evidence in the present data; among HBsAg-positive HCC patients the correlation coefficient is +0.04, whereas among HBsAg-negative HCC patients the corresponding value is +0.05.

### Discussion

The association between $\alpha_1$AT and HCC has been studied from several points of view. Individuals who are homozygous for the Z allele (and perhaps other alleles associated with $\alpha_1$AT deficiency) have an increased risk for HCC, and many of them have the characteristic globular bodies in their livers. However, it is not clear whether individuals who are heterozygous for these alleles are at increased risk for HCC; many studies have noted modest associations but several others have not, even though heterozygotes have consistently the same characteristic globules in their livers (studies reviewed by Sharp, 1976; Kew *et al.*, 1978; Morse, 1978; Kelly *et al.*, 1979; Sizaret *et al.*, 1981; Spech & Liehr, 1982). Kew *et al.* (1978) have summarized the evidence by stating that $\alpha_1$AT deficiency could be an occassional cause of the tumour in most parts of the world where HCC occurs sporadically, but it is not a numerically important cause in Africa and the Far East where HCC is common. Our findings support this conclusion and permit its generalization to other populations where HCC is common, besides those of Africa and Asia. On the other hand, we did not confirm the associations of HCC with the F or the $M_2$ alleles, which were reported by Theodoropoulos *et al.* (1976) and Sizaret *et al.* (1981), respectively. Since the present study is larger than the other two we are inclined to believe that the earlier findings were, perhaps, fortuitous.

Elevated values of serum $\alpha_1$AT in HCC cases have been noted by Kew *et al.*, (1978), Chio & Oon (1979), and Matsuzaki *et al.* (1981). Our findings confirm the results of the earlier investigations and indicate that the elevation is sufficiently marked to be aetiologically intriguing and diagnostically useful. Furthermore, we have found that serum $\alpha_1$AT values are, on the average, higher in HBsAg-negative cases of HCC than in HBsAg-positive cases of this tumour—an observation not reported previously. Although the difference is not sufficiently large to be of clinical importance it suggests that the aetiologic heterogeneity of HCC is reflected not only in the epidemiologic parameters but also in the laboratory findings of the aetiologic subgroups.

The molecular, biochemical and histological aspects of the association between $\alpha_1$AT and HCC have been reviewed elsewhere (Sharp, 1976; Morse, 1978; Palmer *et al.*, 1980; Spech & Liehr, 1982). However, the differential increase of $\alpha_1$AT in HBsAg-positive and HBsAg-negative HCC, if real, calls for explanation. Several possibilities exist. Tobacco smoking has been found to increase the serum levels of $\alpha_1$AT by $\sim$20% (Lellouch *et al.*, 1979) and this proportional excess may also concern the tobacco related HCC cases. It should be noted, also, that cirrhosis is associated with reduced levels of serum $\alpha_1$AT (Chio & Oon, 1979; Matsuzaki *et al.*, 1981), and cirrhosis (in Greece, at least) is more frequently associated with HBsAg-positive than with HBsAg-negative HCC (Trichopoulos *et al.*, 1982).

Palmer *et al.* (1980), using ultrastructural, histochemical and immunocytochemical methods have demonstrated the occasional parallel emergence of $\alpha$FP and $\alpha_1$AT as tumour tissue markers in malignant hepatoma (HCC). It is, therefore, surprising that a significant positive association was not evident between the serum values of $\alpha$FP and $\alpha_1$AT in the present study or in the earlier study of Chio & Oon (1979). Although chance is a likely explanation for the absence of a significant association, it is also possible that the epigenetic emergence of $\alpha_1$AT as tumour tissue marker is not frequently accompanied by an increase in the serum levels of $\alpha_1$AT (Palmer *et al.*, 1980).

Supported by a grant from the Greek Ministry of Health.

### References

CHAPUIS-CELLIER, C. (1975). *Etude biochimique et genetique de l'alpha-1-antitrypsine humaine*. M.D. Thesis, Université Claude Bernard, Lyon, France.

CHAPUIS-CELLIER, C. & ARNAUD, P. (1979). Preferential transmission of the Z deficient allele of alpha-1 antitrypsine. *Science*, **205**, 407.

CHIO, L.F. & OON, C.J. (1979). Changes in serum alpha$_1$ antitrypsin, alpha$_1$ acid glycoprotein and beta$_2$ glycoprotein in patients with malignant hepatocellular carcinoma. *Cancer*, **43**, 596.

COX, D.W. (1978). Genetic variation of alpha-1-antitrypsin. *Am. J. Hum. Genet.*, **30**, 660.

HARPEL, P.C. (1983). Protease inhibitors—a precarious balance. *N. Engl. J. Med.*, **309**, 725.

KELLY, J.K., DAVIES, J.S. & JONES, A.W. (1979). Alpha-1-antitrypsin deficiency and hepatocellular carcinoma. *J. Clin. Pathol.*, **32**, 373.

KEW, M.C., TURNBULL, R. & PRINSLOO, I. (1978). a$_1$-antitrypsin deficiency and hepatocellular cancer. *Br. J. Cancer*, **37**, 635.

LAM, K.C., YU, M.C., LEUNG, J.W.C. & HENDERSON, B.E. (1982). Hepatitis B virus and cigarette smoking-risk factors for hepatocellular carcinoma in Hong-Kong. *Cancer Res.* **42**, 5246.

LELLOUCH, J., CLAUDE, J.R. & THEVENIN, M. (1979). a$_1$-antitrypsin et tabac, une etude de 1296 hommes sains. *Clin. Chim. Acta*, **95**, 337.

MATSUZAKI, S., IWAMURA, K., ITAKURA, M., KAMIGUCHI, H. & KATSUNUMA, T. (1981). A clinical evaluation of serum alpha-1-antichymotrypsin levels in liver disease and cancers. *Gastroenterol. Jpn.*, **16**, 582.

MORSE, O.J. (1978). Alpha$_1$-antitrypsin deficiency. *N. Engl. J. Med.*, **299**, 1045.

PALMER, P.E., UCCI, A.A. & WOLFE, H.J. (1980). Expression of protein markers in malignant hepatoma. *Cancer*, **45**, 1424.

SHARP, H.L. (1976). The current status of a-1-antitrypsin, a protease inhibitor, in gastrointestinal disease. *Gastroenterology*, **70**, 611.

SIZARET, P., CLERC, M., ESTÈVE, J., FRANTS, R.R. & PILLOT, J. (1981). M$_2$ alpha-1-antitrypsin phenotype and primary liver cancer. *Br. J. Cancer*, **43**, 226.

SPECH, H.J. & LIEHR, H. (1982). Alpha 1-antitrypsin deficiency and the liver. *Z. Gastroenterol.*, **20**, 393.

THEODOROPOULOS, G., FERTAKIS, A., ARCHIMANDRITIS, A., KAPORDELIS, C. & ANGELOPOULOS, V. (1976). Alpha-1-antitrypsin phenotypes in cirrhosis and hepatoma. *Acta Hepatogastroenterol.*, **23**, 114.

TRICHOPOULOS, D., KREMASTINOU, J. & TZONOU, A. (1982). Does Hepatitis B Virus Cause Hepatocellular Carcinoma? In *Host Factors in Human Carcinogenesis*, p. 317 (Ed. Bartsch & Armstrong). International Agency for Research on Cancer/Commission of the European Communities. IARC Scientific Publication No. 39: Lyon.

TRICHOPOULOS, D., MACMAHON, B., SPAROS, L. & MERIKAS, G. (1980). Smoking and Hepatitis B-negative Primary Hepatocellular Carcinoma. *J. Natl. Cancer Inst.* **65**, 111.

TRICHOPOULOS, D., TABOR, E., GERETY, R.J., XIROUCHAKI, E., SPAROS, L., MUNOZ, N. & LINSELL, C.A. (1978). Hepatitis B and Primary Hepatocellular Carcinoma in a European Population. *Lancet*, **ii**, 1217.

VESTERBERG, O. (1973). Isoelectric focusing of proteins in thin layers of polyacrylamide gel. *Science Tools*, **20**, 22.